# Determination of French influenza outbreaks periods between 1985 and 2011 through a web-based Delphi method

**DOI:** 10.1186/1472-6947-13-138

**Published:** 2013-12-24

**Authors:** Marion Debin, Cécile Souty, Clément Turbelin, Thierry Blanchon, Pierre-Yves Boëlle, Thomas Hanslik, Gilles Hejblum, Yann Le Strat, Flavien Quintus, Alessandra Falchi

**Affiliations:** 1 Institut National de la Santé et de la Recherche Médicale, UMR-S 707, F-75012 Paris, France; 2 Sorbonne Universités, UPMC Univ Paris 06, UMR-S 707, F-75012 Paris, France; 3 Assistance Publique-Hôpitaux de Paris, Hôpital Saint-Antoine, Unité de Santé Publique, F-75012 Paris, France; 4 Université Versailles Saint Quentin en Yvelines, F-78000 Versailles, France; 5 Département des maladies infectieuses, Institut de Veille Sanitaire (InVS), F-94415 St Maurice, France; 6 Réseau Sentinelles, U707 Inserm, Université de Corse, Laboratoire de Virologie, F-20250 Corte, France

**Keywords:** Delphi technique, Information science, Consensus, Influenza, Epidemics, Surveillance

## Abstract

**Background:**

Assessing the accuracy of influenza epidemic periods determined by statistical models is important to improve the performance of algorithms used in real-time syndromic surveillance systems. This is a difficult problem to address in the absence of a reliable gold standard. The objective of this study is to establish an expert-based determination of the start and the end of influenza epidemics in France.

**Methods:**

A three-round international web-based Delphi survey was proposed to 288 eligible influenza experts. Fifty-seven (20%) experts completed the three-rounds of the study. The experts were invited to indicate the starting and the ending week of influenza epidemics, on 32 time-series graphs of influenza seasons drawn using data from the French *Sentinelles* Network (Influenza-like illness incidence rates) and virological data from the WHO-FluNet. Twenty-six of 32 time-series graphs proposed corresponded to each of the French influenza seasons observed between 1985 and 2011. Six influenza seasons were proposed twice at each round to measure variation among expert responses.

**Results:**

We obtained consensual results for 88% (23/26) of the epidemic periods. In two or three rounds (depending on the season) answers gathered around modes, and the internal control demonstrated a good reproducibility of the answers. Virological data did not appear to have a significant impact on the answers or the level of consensus, except for a season with a major mismatch between virological and incidence data timings.

**Conclusions:**

Thanks to this international web-based Delphi survey, we obtained reproducible, stable and consensual results for the majority of the French influenza epidemic curves analysed. The detailed curves together with the estimates from the Delphi study could be a helpful tool for assessing the performance of statistical outbreak detection methods, in order to optimize them.

## Background

The primary objective of sentinel surveillance is to provide sensitive, specific, and timely alerts at the beginning of increased disease activity. Timely detection of the start and the end of influenza outbreaks is crucial for a number of reasons such as improving communication towards persons eligible for vaccination, better planning of the organization of the health care system and estimating the vaccine effectiveness [1,2].

The French *Sentinelles* Network (http://www.sentiweb.fr/?page=database) [3,4], like other surveillance systems [5-7], uses an approach based on a seasonal regression model proposed by Serfling for influenza outbreak monitoring [8]. Under this model, influenza-like illness (ILI) incidence rates from non-epidemic weeks in the previous years are used to compute a time-varying threshold. A statistical alarm is triggered if weekly incidence rate exceeds this threshold [9]. The influenza outbreak is publicly declared when the threshold is exceeded during two consecutive weeks [10]. Since no mathematical definition of an outbreak exists, assessing the value of the estimates from models requires a reliable gold standard, which define at what time a given outbreak has begun and ended in reality. Such a gold standard is currently not available.

Our objective is to address this absence of a gold standard by establishing an expert-based determination of influenza epidemic periods through a Delphi process [11]. This method involves the anonymous completion of a questionnaire on several occasions and has been proposed for eliciting the optimal decision in a problem where decision-making is not straightforward [12,13].

We undertake an international web-based Delphi survey among influenza experts, invited to indicate the starting and the ending week of influenza epidemics, on graphs of influenza seasons drawn using data from the French *Sentinelles* Network (ILI incidence rates) and virological data from the WHO-FluNet.

## Methods

### The web-based Delphi process deployment

**Figure 1:**
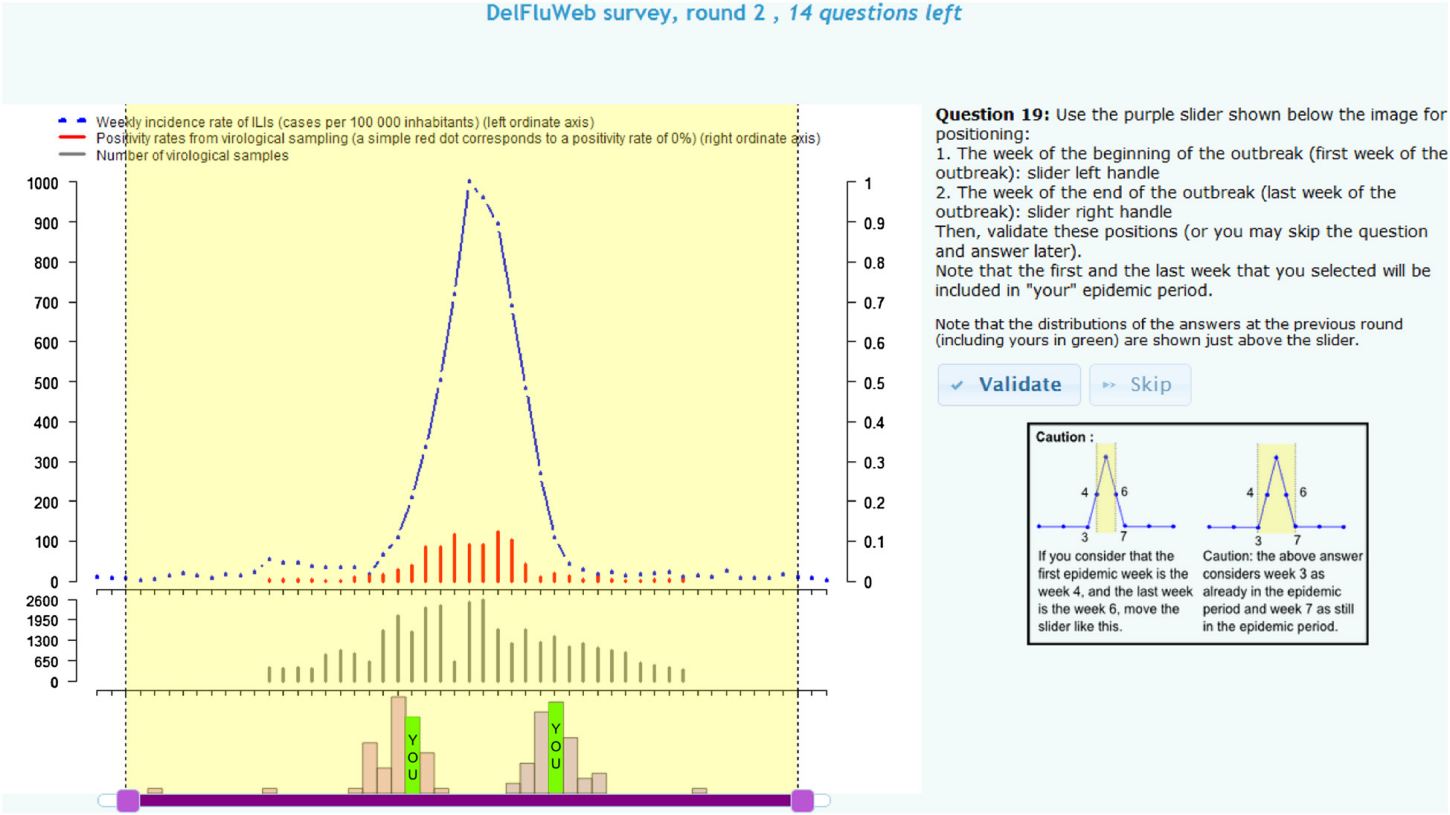
**A typical question screen proposed to the experts during the completion of the questionnaire.** The computer application provided epidemic curves to experts with an associated user-friendly slider (purple color, bottom left), the latter, with its two extreme handles, enabling experts to easily position the corresponding beginning and end of the outbreak. Just above the slider, a bar plot displaying the distribution of the answers collected at the previous round is provided as a feedback, with the own answer of the expert highlighted in a different (green) colour. The right part of the Internet page displays guiding information.

The Delphi method involves the anonymous completion of a questionnaire presented to a panel of experts on successive occasions, called rounds. In a typical Delphi study, during the first round, each expert fills in the questionnaire. During subsequent rounds, they are again invited to fill in the questionnaire but they are also allowed to alter their initial choices, at the light of a provided feedback on the previous round responses of the group (as shown in Figure 1) [11].

This study was deployed using a previously PHP/MySQL-based computer application devoted to online Delphi surveys [14]. We further developed this application to display influenza epidemic curves as illustrated in Figure 1.

### Ethics statement

The protocol was conducted in agreement with the Helsinki declaration and was approved by the ethical committee (CPP Ile de France V). We obtained authorization from the French Data Protection Agency (CNIL, registration number #471393) covering all non-publicly available data included in this study.

### Selection of the expert panel

Four complementary sources were used to build the list of experts solicited in the study: network members of WHO Euroflu (key officials actively involved in European influenza surveillance) [15], North-American experts selected by an American influenza expert belonging to the National Institutes of Health, members of the “French influenza working group” within “French Public Health Council” (HCSP), and influenza experts selected via a systematic research on Medline [16].

An information sheet describing the proposed study and inviting to participate was emailed to the 322 potential participants. On each round, reminders were sent to those who did not answer nor declined the invitation.

### Questionnaire

**Figure 2:**
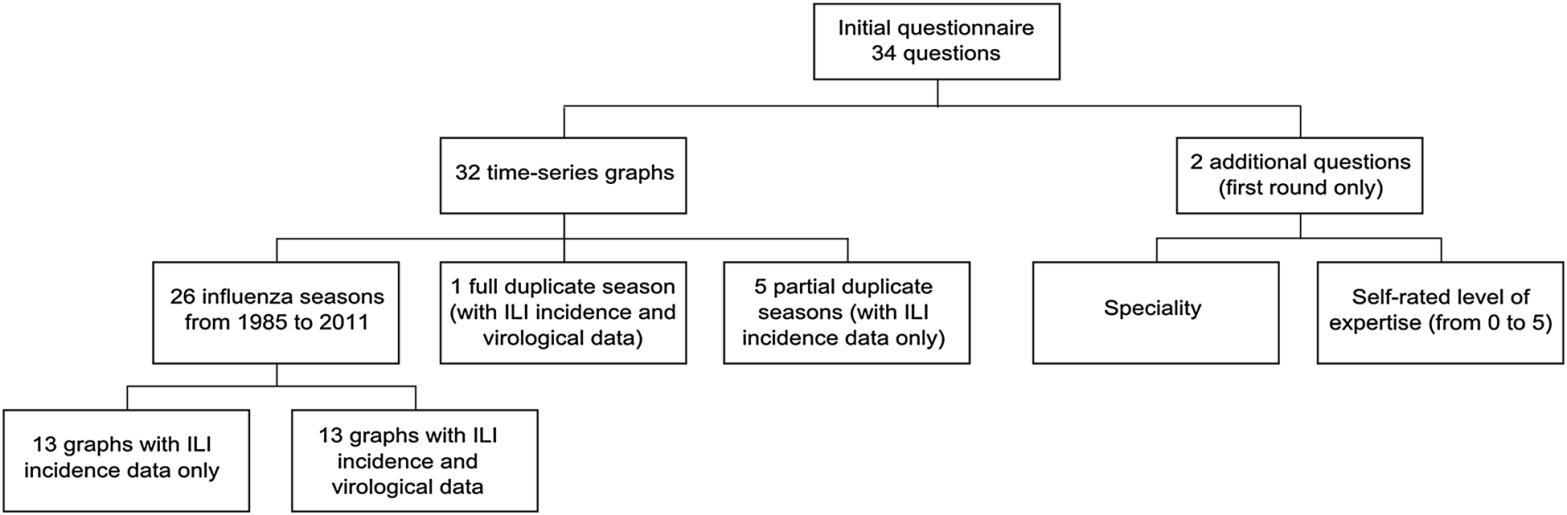
Organisation of the survey.

The first round questionnaire included 34 questions (Figure 2) of which 32 were time-series graphs. On each of the 32 graphs, experts were invited to indicate the starting and the ending week of the influenza epidemic period by using a slider (Figure 1).

**Figure 3:**
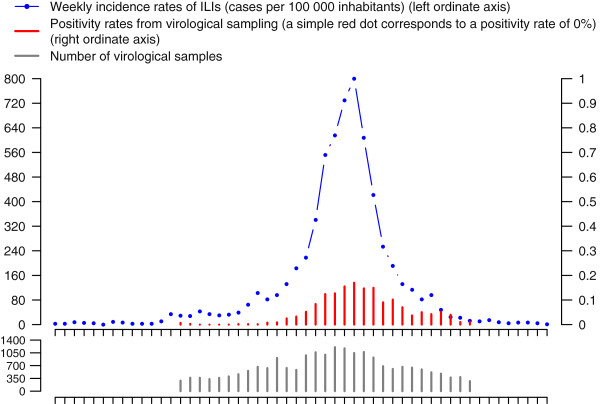
**An epidemic curve standard display.** Weekly national incidence rates of ILI data (blue dashed line) were obtained from the French *Sentinelles* Network. ILI was defined as: sudden fever > 39°C (102°F) with myalgia and respiratory signs. Weekly national proportions of confirmed influenza positive samples (virological data, red bar plot) data were downloaded from WHO-FluNet [17] (data provided by the French National Reference Center for flu, cell culture or PCR test, all influenza virus combined), for mainland France. The grey bar plot at the bottom of the graph corresponds to the number of virological samples analyzed per week.

Each graph included data from the French *Sentinelles* Network (http://www.sentiweb.fr) [3,4] and from WHO-FluNet [17] (Figure 3): weekly national ILI incidence rates, weekly national proportions of confirmed influenza positive samples (virological data, available since 1997–1998), numbers of virological samples analyzed per week. Data were shown from the beginning of July (week 27) of the year to the end of June (week 26) of the following year, called seasons thereafter. Weeks were not numbered on the graphs and no indication to the year was given to the experts.

Among the 32 graphs, 26 corresponded to one of the French influenza seasons from 1985 to 2011 and six showed one of these same graphs but in duplicate (Figure 2), in order to measure variation within experts. Among the six duplicates, five influenza seasons (named *partial duplicate seasons*) were presented twice at each round, but with different information: either both ILI incidence and virological data or only ILI incidence data; and one influenza season (named *full duplicate season*), describing incidence and virological data, was presented two times at each round as internal quality-control tool.

The choice of the *full/partial duplicate season(s)* was random (same years for all experts and all rounds). The order of the 32 graphs was randomly defined for each expert and each round, except for the *duplicate seasons* which positions were non-consecutive, selected at random, and fixed for all experts and all rounds, in order to avoid neighbouring duplicates. To prevent the outstanding 2009–2010 influenza curve (H1N1 pandemic) from altering the assessment of the other epidemic curves, the corresponding graph was the last presented.

Finally, in order to collect the auto-evaluated level of expertise of the participants and their speciality, we added at the end of the first round (Figure 2) the two following questions: *“How would you rate your level of expertise in influenza outbreak detection?”*(answer from “1 - Low level of expertise” to “5 - High level of expertise”) and *“What is your main occupation?”* (multiple choice question - Epidemiologist/Virologist/Modelling specialist/Clinician/Other). The mean level of expertise of the experts who participated in the entire study was compared to the mean level of expertise of the experts who partially participated in the study, by using a Mann–Whitney non-parametric test.

### Number of rounds and stopping criterion

The first round began at the end of May 2012; the third and last round ended in August 2012. A graph was not to be presented at the next round if the answers collected at a given round were stable, as compared to the previous round answers. We considered that such stability was reached whenever at least 75% of the experts had not moved the dates of the beginning and the end of the epidemic period by more than one week (experts named *stable experts*). This stopping criterion was applied to each graph. The study ended when the stopping criterion was reached for every graph.

### Determination of the start and end of influenza epidemics and definition of the level of consensus

As previously proposed [14], because extreme values may correspond to erroneous answers, we eliminated for each graph at each round the 5% lowest values and 5% highest values. Considering the remaining 90% answers, the mode (most often cited answer), the median, the range and the interquartile range (IQR) of each bound (beginning and end of each epidemic period) were calculated. The mode and the median were used for estimating the central tendency of the distribution of the panel’s answers, while the IQR was used as a measure of the variability across the panel answers.

The mode was considered as most informative and was used to define the timing of each influenza outbreak. In the case of several modes, the closest to the median was preferred. The main analysis to define the period of the influenza outbreak concerned the 26 initial graphs. For the *partial duplicate seasons*, the one considered in the main analysis was the graph with virological data.

To define a consensus among respondents, we used the cut off previously used by Norder and al. [18], and defined three levels of consensus as a function of the percentage of experts whose answer was no different than one week from the mode: when this percentage was at least 75%, was between 50% and 75%, and was below 50%, the corresponding level of consensus was categorized as *high*, *medium*, and *low*, respectively. In order to evaluate the answers coherence of each expert we studied the differences between the modes and the individual answers, for each expert, and calculated for each expert the standard deviation (SD) of these differences.

### Reproducibility and influence of virological data

We compared the answers of the *duplicate seasons* together (mode, median, range, IQR). For the *full duplicate season*, we studied, for each expert, if the same answers were given for the two graphs. For the *partial duplicate seasons,* we compared the level of consensus achieved between the graphs with ILI incidence and virological data and the graphs with ILI incidence data only.

All analyses were performed with the R software, version 2.8.1 [19].

## Results

Three rounds were necessary to reach the stopping criterion for every graph. The two first rounds lasted three weeks, and the third round lasted seven weeks. An example of the answers given at each round is shown in Additional file 1.

### Participation rate

The number of participating experts is detailed in Table 1. Of the 322 invitations sent, 288 experts were eligible in the study, and 69 (24%) participated in round 1. Sixty-one of 69 (88%) participated in the second round, and 57 (83%) in the third round. The participation rates were of 21% (61/288) and 20% (57/288) in the second and third round, respectively. The mean level of expertise of the experts who partially participated in the study did not differ from the mean level of expertise of the experts who participated in the three rounds (3.6 vs. 3.7, p = 0.87). The mean level of expertise of the 61 experts who answered to this question was 3.7 (median = 4, SD = 0.9). Among them, 1 expert (2%) self-rated with 1 (low level of expertise), 3 (5%) with 2, 20 (33%) with 3, 27 (44%) with 4 and 10 (16%) with 5 (high level of expertise). At the first round, the panel was composed of 34 epidemiologists (57%), 17 virologists (28%), 4 clinicians (6%), 2 modelling specialists (3%) and 4 other specialities (6%). Modelling specialists reported the highest level of expertise (mean = 4.0, standard deviation = 1.4). Both epidemiologists (SD = 0.7) and virologists (SD = 0.8) self-rated with a mean level of 3.8, clinicians with 2.2 (SD = 1.3) and other specialities with 3.5 (SD = 0.6). The experts originated from 33 countries: 10 from France, 39 from other European countries, 9 from North America, 2 from Central Asia and 1 from Eastern Asia.

**Table 1:** Number of experts who participated in the study, depending on the way of selection

	**Number of invited experts**	**Non eligible experts; erroneous email addresses**	**Number of eligible experts**	**Number of participating experts in round 1 (% of eligible experts)**	**In round 2 (% of round 1)**	**In round 3 (% of round 1)**
Euroflu contact points	220	3; 14	203	48 (24)	42 (88)	39 (81)
North-American experts	9	0; 0	9	5 (56)	5 (100)	4 (80)
French influenza working group	24	2; 1	21	5 (24)	3 (60)	3 (60)
Literature research (Medline®)	69	0; 14	55	11 (20)	11 (100)	11 (100)
Total	322	5; 29	288	69 (24)	61 (88)	57 (83)

### Stopping criterion

At the end of the second round, the stopping criterion was reached for 19 (59%) of the 32 graphs presented (mean proportion of *stable experts* for these graphs = 82%). At the end of the third round, the stopping criterion was obtained for the 13 remaining graphs (mean proportion of *stable experts* = 84%).

### Determination of the start and end of each outbreak and level of consensus

The detailed results are available in Table 2 and in Additional file 2, and the complete database of the study is available upon request. Overall, a *high level of consensus* was achieved for 20 beginning bounds (77%) of the 26 initial graphs, and for 15 ending bounds (58%). The mean IQR of the answers was 1.8 weeks (1.5 weeks for beginning bounds, 2.0 weeks for ending bounds). We obtained consensual results for 88% (23/26) of epidemic periods. A *high level of consensus* was obtained for the beginning and the end of 12 epidemic periods in 26 (46%). For 10 epidemic periods (38%), a *high level of consensus* was obtained for one bound, and a *medium level of consensus* for the other bound. For one epidemic period (4%) the consensus was *medium* for the two bounds, and for three epidemic periods (12%) there was a *low level of consensus* for one of the bounds or for the two bounds.

**Table 2:** Level of consensus obtained at the end of the study

	**Number of seasons**	**Nb of seasons with a *high level of consensus *** **for the starting bound (%)**	**Nb of seasons with a *high level of consensus *** **for the ending bound (%)**	**Nb of seasons with a *high level of consensus *** **for the starting and ending bound (%)**	**Mean IQR (in weeks)**
All seasons	26	20 (77)	15 (58)	12 (46)	1.8
Seasons with ILI incidence and virological data	13	10 (77)	7 (54)	5 (38)	1.8
Seasons with ILI incidence data only	13	10 (77)	8 (62)	7 (54)	1.7

The mean IQR of the answers was 2.8 weeks on round 1, 1.8 weeks on round 2 and 2.1 weeks on round 3. The SD per expert of the differences between the modes and the individual answers ranged between 0.0 week and 11.1 weeks, with a mean of 2.5 weeks (median = 1.7 week, IQR = 2.2).

### Reproducibility of the answers

For the two *full duplicate* graphs presented (internal quality-control tool), the stopping criterion was achieved at the end of the second round. On the first round, 34 experts in 61 who answered these two questions (56%) gave the same answers for the two graphs, 24 experts (39%) gave the same answers more or less one week for the beginning and the end of the epidemic period, and 3 experts (5%) gave answers differing from more than one week for the beginning or the end of the epidemic period. During the second round, they were respectively 39 in 60 (65%), 19 (30%) and 3 (5%). At the end of the second round, the median, the mode and the range of the answers were the same for the two graphs.

### Influence of virological data

At the last round where each graph was presented, the mode and the median were the same for three of the five *partial duplicate seasons*. For one influenza season (2006–2007), the medians were slightly different (0.5 week of difference). For one influenza season (1998–1999), the modes and medians were both different between the two graphs (difference of one and two weeks respectively).

The level of consensus achieved for the graphs with only ILI incidence data and for the graphs with ILI incidence and virological data was not different for the beginning bounds (*high level of consensus* achieved for three out of five graphs) and for both bounds (one out of five graphs) of the *partial duplicates.* For the ending bounds, a high level of consensus was achieved for respectively three out of five and two out of five graphs. The mean IQR was 1.6 weeks for the graphs with only ILI incidence data and 2 weeks for the graph with ILI incidence and virological data.

## Discussion

In the present study, we describe the usefulness of the Delphi method to establish the dates of the start and the end of 26 influenza epidemics. Thanks to this international web-based Delphi survey among influenza experts, we obtained reproducible, stable and consensual results, for 23 of the 26 French influenza epidemic curves analysed. The provided detailed curves together with the estimates issued from the Delphi study constitute a helpful tool for assessing the performance of statistical outbreak detection methods, in order to optimize them.

### Determination of the start and end of influenza epidemics

Median is commonly used to summarize results in Delphi studies, given the non-continuous nature of the answers, and the expected skewness of the distribution of the answers [20]. The selection criterion chosen to summarize the experts’ answers and define the period of each influenza outbreak was a week number. In this study, the median could correspond to a value not chosen by any expert, if half of the experts answered that the date was before and half answered that the date was after, and we decided to use the mode. It is noteworthy that in the present study, we observed that 77% of the bounds of the epidemic period have a mode equal to the median.

One of the possible issues of this study could have been a difference in the dates obtained with the *duplicate seasons*. The period of influenza outbreak determined by the study were the same for the two graphs of the *full duplicate season* and for the graphs of the *partial duplicate seasons*, except for the season 1998–1999. For this season, there was a two weeks difference between the two graphs (with or without virological data) for the beginning of the season, and a one week difference for the end of the season. As shown in Additional file 3 there was a 2-week lag between the increase (≥150% from the previous week) of the ILI incidence rate and the increase of the positivity rates from virological samplings. The virological positivity rates varied a lot during this influenza season, making it very difficult to interpret.

### Level of consensus

The selection criteria most often used in Delphi studies are the validity, the feasibility, the agreement or the importance of several statements [21]. To collect the views of the experts, scales are often used, like the 9 or 5-point Likert scale [21]. Therefore, the analyses aim at determining whether the panel agrees or not with each statement of the questionnaire [12]. To compute the level of consensus achieved, the several possible answers are frequently grouped together, in order to obtain only three groups of answer: “disagree”, “uncertain”, “agree”. As we wanted here to determine a date, and not a global “agreement” or “disagreement” we grouped the answers around the mode (from one week before to one week after) to determine if experts agree or not with this answer. This method increases the agreement rate measured within experts, but do not affect the selected date. For the *full duplicate season*, the answers given by the experts were very similar, which shows the good reproducibility of the answers. Nevertheless, a mean difference of 0.4 week for the beginning bounds and 0.2 week for the ending bounds were observed, showing that for a same graph and a same expert, the answers can slightly differ, and that it is safe to include a ± one week window around the mode in the calculation of the level of consensus.

The next critical point is the determination of a cut off to define the level of consensus achieved. In line with several previous works, we considered a 75% cut off, but no scientific observations supported that choice [18,22-24]. Many other cut off have been used in previous Delphi studies to define a consensus: 51% agreement amongst respondents [25,26], 60% agreement [27], 70% agreement [28], 80% agreement [29,30]. We have chosen a cut-off located in the “mid-range” of the values found in literature. In our case, the goal was not to obtain a total agreement on all epidemic periods, but to obtain beginning and ending dates for each epidemic period, the definition of the level of the consensus was therefore not crucial.

### Influence of virological data

The slight difference observed for the *partial duplicate seasons* (with or without virological data) suggests that the presence of virological data have a small impact on the stability of the answers and the degree of consensus reached. Virological data appear as having little informative value on the determination of the period of each influenza outbreak.

### Limitations of the study

One of the limitations of the study is the possible issues in understanding the protocol, emphasized by the fact that English was not the native language of most of the experts. For example, a few experts have studied the graphs “in real time” (as if they were studying the data during the influenza season and had to declare the beginning of the epidemic), and not in retrospect as we expected, which leads to a difference in the way of interpreting the curves and answering the questions. Anyway, we believe that eliminating the 10% of extreme answers allowed us to get rid of most of the erroneous answers due to a comprehension issue.

Another limitation of the study could be the variability in the given answers. The mean IQR of the answers decreased from 2.8 weeks on round 1 to 1.8 weeks on round 2, a classical finding that could indicate that the consensus between experts increased [21]. As expected, because in the third round only the graphs with a high variability of answers between the two first rounds were presented to experts, the mean IQR was higher for the third round (mean IQR = 2.1). The SD of the differences between the modes and individual responses per expert was rather small, indicating that most of the experts mainly gave their answers right before or after the mode. Response variability was mainly due to the variability between experts, which improves the relevance of our results.

The response rate of our study was 24% (69/288) in the first round, 20% (57/288) in the last round. Few articles on Delphi studies reported their response rate for all rounds (39% of 80 articles studied by Boulkedid and al.), which biases the comparison, but when this rate is reported, the median response rate is 88% in the last round [21]. However, experts are frequently asked first about their willingness to participate, in order to improve the raw number of participants, which was not done here. This can partially explain our low response rate. The total number of participating experts (57 experts) was nevertheless quite high, when compared to the mean number of individuals invited to participate in the 80 studies reviewed by Boulkedid and al. [21] (17 people, Q1 = 11, Q2 = 31). The low dropout rate (17%) was very satisfying, as a high dropout rate may lead to a non representative subgroup of the original population of experts, and bias the results [31,32]. Furthermore, the experts who participated partially in the study did not differ from the one who participated in the entire study in term of self-rated level of expertise. According to the practical guidance elaborated by Boulkedid and al. [21], it is recommended to create a heterogeneous group of experts (with different specialities), and invite a large number of experts, if possible from different countries. We therefore decided to give priority to the number of experts and the heterogeneity of the group, inviting many people from different countries, without asking them first about their willingness to participate.

The protocol and the analysis of this study were hardly supported by previous work, because of the absence of comparable studies in literature. Several indicators nevertheless demonstrate that the results were very satisfying and may be used to evaluate and improve statistical outbreak detection methods.

## Conclusion

This study shows that it is possible to use a web-based Delphi procedure with the aim of determining the period of past influenza outbreaks. In two or three rounds (depending on the season) answers gathered around a mode, and the internal control demonstrated a good reproducibility of the given answers. It was shown that the presence of virological data do not have a significant impact on the answers, except for a season with a major mismatch between virological and incidence data timings. The results of this study will be used for further statistical works aiming to improve statistical outbreak detection methods.

## Competing interests

The authors declare that they have no competing interests.

## Authors’ contributions

MD and AF were involved in collecting the data, analysis of data and preparation of manuscript. CS and CT were involved in the analysis of the data and in technical aspects of the project. FQ was involved in the development of the website. TB, TH, GH, PYB, YLS were involved in the conception and coordination of the study. The DelFluWeb study group answered to the three rounds of the Delphi questionnaire. All authors read and approved the final manuscript.

## Pre-publication history

The pre-publication history for this paper can be accessed here:

http://www.biomedcentral.com/1472-6947/13/138/prepub

## Supplementary Material

Additional file 1 **Summary of the results given at the three rounds for one of the graphs.** The answers given by all the experts at each round for the beginning (green) and the end of the outbreak (orange) are summarized in the bar chart (number of experts who answered this week). The medians of the answers are indicated with a dashed line, and the mode is indicated with a colored bar. This graph corresponds to the 2000–2001 influenza season. Click here for file

Additional file 2 **Summary of the results, at the last round where each graph was presented.** In the first column, in bold appear the years of the graphs with a *high level of consensus* for the starting and ending bounds. In the 6th and 10th columns, in bold appear the bounds with a *high level of consensus*. All data used for building the graphs shown during this Delphi can be downloaded on the website of the French *Sentinelles* Network (http://websenti.u707.jussieu.fr/sentiweb/?page=database) and the website of Flunet (http://apps.who.int/globalatlas/dataQuery/default.asp). * Graphs not shown at round three. Click here for file

Additional file 3 The graph of the 1998–1999 influenza season. Click here for file
